# New Properties of a Well-Known Antioxidant: Pleiotropic Effects of Human Lactoferrin in Mice Exposed to Gamma Irradiation in a Sublethal Dose

**DOI:** 10.3390/antiox11091833

**Published:** 2022-09-18

**Authors:** Marina Yu. Kopaeva, Irina B. Alchinova, Anton B. Cherepov, Marina S. Demorzhi, Mikhail V. Nesterenko, Irina Yu. Zarayskaya, Mikhail Yu. Karganov

**Affiliations:** 1 National Research Center “Kurchatov Institute”, 1 Akademika Kurchatova Sq., 123182 Moscow, Russia; 2 Institute of General Pathology and Pathophysiology, 8 Baltiyskaya St., 125315 Moscow, Russia; 3 “Lactobio” LLC, 29 Prospekt Vernadskogo, 119331 Moscow, Russia

**Keywords:** human lactoferrin, acute gamma irradiation, C57Bl/6 mice, survival rate, open field test, spleen, serum homeostasis, leukocytes

## Abstract

We studied the effects of human lactoferrin (hLf), a multifunctional protein from the transferrin family, on integral (survival, lifespan during the experiment, body weight, behavior, subfractional compositions of blood serum) and systemic (hemoglobin level, leukocyte number, differential leukocyte count, histological structure of the liver and spleen) parameters of the body in mice after acute gamma irradiation in a sublethal dose. The experiments were performed on male C57BL/6 mice. The mice in the experimental groups were exposed to whole-body gamma radiation in a dose of 7.5 Gy from a ^60^Co source. Immediately after irradiation and 24 h after it, some animals received an intraperitoneal injection of hLf (4 mg/mouse). Single or repeated administration of hLf had a positive pleiotropic effect on irradiated animals: animal survival increased from 28% to 78%, and the mean life expectancy during the experiment (30 days) increased from 16 to 26 days. A compensatory effect of hLf on radiation-induced body weight loss, changes in homeostasis parameters, and a protective effect on the structural organization of the spleen were demonstrated. These data indicate that Lf has potential as a means of early therapy after radiation exposure.

## 1. Introduction

Ionizing radiation is a phenomenon that is present in our daily life, originating from both natural and artificial sources. The power of this radiation can be either within the natural background range or significantly exceed it. During radiation therapy, one of the leading therapeutic methods of treating many types of human cancer, as well as during radiodiagnosis, the patients can develop side effects, including immunosuppression, changes in blood composition, and mucosal lesions. Medical workers in radiological laboratories and X-ray rooms are occupationally exposed to radiation and are at risk of developing the same adverse effects. The interaction of pathogenetic and adaptive processes underlying these adverse effects and activated by radiation exposure occurs at all levels of biological organization—from molecules to the whole body. In this context, the search for effective therapeutic agents of pleiotropic action for the treatment of socially significant diseases associated with radiation exposure is an urgent problem.

According to existing ideas about the pathogenesis of radiation damage, a leading role in the mechanism of its development is played by oxidative stress [1,2]. Reactive oxygen species and free radicals can initiate long-lasting alternative processes in various organs and tissues. The destructive effect of free radicals can be prevented by antioxidants that reduce the damaging effect of ionizing radiation on the body. Their protective effect is related to the suppression of free radical oxidation and activation of the antioxidant systems in the body [3]. One of these antioxidants, protein lactoferrin, inhibits the Fenton reaction through iron chelation [4,5].

Lactoferrin (Lf) is a multifunctional globular glycoprotein with a molecular weight of ~80 kDa, a member of the transferrin family. It is present in various mammalian secretory fluids, as well as in neutrophil granules [6,7]. Lf is involved in various physiological processes, including binding and transport of iron ions and immune and inflammatory reactions. This protein has multiple protective functions [8] and radioprotective properties [9]. The biological effects of Lf are mediated by specific surface receptors on target cells. Lf receptors were detected on B cells, T cells, monocytes [10,11], platelets [12], hepatocytes [13], fibroblasts [14], osteoblasts [15], and endothelial cells of brain vessels [16].

There are several options for drugs to reduce radiation damage. Nevertheless, current treatments, although beneficial, may have attendant side effects and long-term sequelae, usually more or less affecting the quality of patients’ lives. Lf shows high bioavailability after oral, intravenous, intranasal, and intraperitoneal administration; high selectivity toward damaged cells; and a wide range of molecular targets controlling cell proliferation, survival and migration [17]. Moreover, Lf can prevent or inhibit the development of radiation damage by stimulation of the adaptive immune response [9].

Numerous tests in animals and humans have proven Lf safety and tolerability. No adverse effect was observed in both rats orally administered with Lf at 2000 mg/kg per 13 days [18] and in humans with 1.5–9 g of Lf per 15 days [19]. There were no changes attributable to administration of the Lf in clinical signs, body weight, food consumption, ophthalmological findings, blood chemistry, or gross pathological examination findings. No hematological, hepatic, or renal toxicities were reported. The lack of toxicity was observed in previous studies in healthy volunteers with doses at 15 g in a 24-h period [20], and no toxicities were found in mouse with doses at 1000 mg/kg administered twice daily for 8 days [21]. In addition to oral administration, several different other modes were tested in animal models and humans. No adverse effect was observed in mice intravenously administered with Lf at 250 μg/g [22]. Of note, Lf is classified as a “generally recognized as safe” (GRAS) substance by the USA Food and Drug Administration [23].

The therapeutic effect of the chronic administration of bovine Lf after whole-body exposure to X-rays was demonstrated; it manifested itself in increasing the survival rate of irradiated mice, stimulating hematopoiesis [4,24], and reducing damage to the small intestinal epithelium [25]. The use of Lf as a therapeutic agent to minimize radiation-induced damage remains rarely studied, and therefore, a comprehensive study of the effect of Lf on the dynamics of the state of the body is required.

Our aim was to study the effects of human Lf (hLf) on integral (survival, lifespan during the experiment, body weight, behavior, subfractional compositions of blood serum) and systemic (hemoglobin level, leukocyte number, differential leukocyte count, histological structure of the liver and spleen) indicators of the organism after acute gamma irradiation of mice in a sublethal dose. Lf was administered to animals once or twice, unlike most previous studies, where long-term repeated administration of Lf was used. The study included a wide range of methods at the body, organ, tissue, cellular, and molecular levels.

## 2. Materials and Methods

The study was conducted on 2–2.5-month-old mice (*n* = 238) weighing 20–28 g (Pushchino nursery for laboratory animals, a branch of the M. M. Shemyakin and Yu. A. Ovchinnikov Institute of Bioorganic Chemistry, Russian Academy of Science). The animals were kept in standard laboratory cages, type 1285L (Techniplast, Buguggiate, Italy), five mice per cage, at controlled temperature and humidity, 12/12 h light–dark cycle with free access to food and water. All experimental procedures were carried out during the light hours between 9:00 and 18:00 h. All manipulations were carried out in accordance with Directive 2010/63/EU on the Protection of Animals Used for Scientific Purposes [26]; the study was approved by the Local Ethical Committee on Biomedical Research of the National Research Center “Kurchatov Institute” (Protocol No. 1, 13 February 2020) and Ethical Committee of the Institute of General Pathology and Pathophysiology (Protocol No. 4, 7 October 2021).

We used hLf (Lactobio LLC, Moscow, Russia) isolated from female colostrum by preparative ion exchange chromatography [27] and additionally purified by affinity chromatography on heparin-sepharose [28]. HPLC by the method of Y. Zhang [29] showed that the purity of the resulting protein preparation was 97%.

The animals were exposed to gamma radiation from a ^60^Co source (GUT-200M apparatus; National Research Center “Kurchatov Institute”, Moscow, Russia). The mice were weighed every three days throughout the experiment on electronic scales Adventurer Pro (Ohaus Corporation, Pine Brook, NJ, USA). The effect of hLf on animal behavior was evaluated using the open field test. Changes in physiological parameters were assessed by shifts in the subfractional composition of the blood plasma assessed by the dynamic light scattering method, total and differential leukocyte count, hemoglobin level, and histological examination of the liver and spleen at different terms after irradiation. Three independent experimental series were performed.

### 2.1. Experimental Groups and Treatments

The mice were randomly divided into seven groups: experimental (IR, *n* = 50; IR+Lf, *n* = 44; IR+Lf×2, *n* = 45), active control (sham-irradiated: AC, *n* = 28; AC+Lf, *n* = 28; AC+Lf×2, *n* = 27), and passive control (intact: PC, *n* = 16). The mice in the experimental groups were exposed to whole-body gamma radiation in a dose of 7.5 Gy (at a dose rate of 0.6 Gy/min). Exposure to this sublethal dose induces serious changes in behavioral and physiological parameters in mice but allows us to keep alive a sufficient number of animals for analysis during a 30-day experiment [30,31].

**Figure 1:**
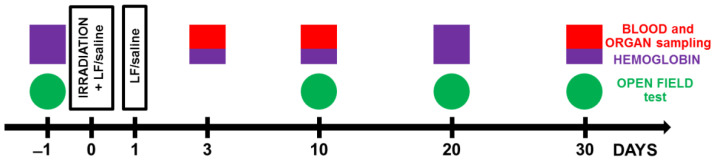
Experimental timeline. Animals from experimental groups were exposed to 7.5 Gy whole-body gamma irradiation. Some animals received intraperitoneal injections of Lf (4 mg/mouse) immediately and 24 h after irradiation. The open field test (OF) was performed prior to irradiation and on days 10, 20, and 30 after it. On days 3, 10, and 30 after irradiation, organs (liver, spleen) and whole blood were taken for analysis. The hemoglobin level and the number of leukocytes in the peripheral blood of animals were determined.

Immediately after irradiation, the animals in groups IR+Lf, IR+Lf×2, AC+Lf, and AC+Lf×2 received intraperitoneal injection of hLf (4 mg/mouse in 0.3 mL of saline (0.9% NaCl, Dalkhimpharm, Khabarovsk, Russia)). In 24 h after irradiation, the mice were re-injected with hLf (groups IR+Lf×2, AC+Lf×2) or saline (groups IR+Lf, AC+Lf). The dose of Lf was chosen based on published data [4], results of our previous studies [32,33], and pilot experiments [34]. The animals in the IR and AC groups were twice injected with saline in the same way. The scheme of the experiment is shown in Figure 1.

### 2.2. Analysis of Mouse Behavior in the Open Field Test

The open field test (OF) is a standard method for assessing spontaneous motor activity and behavior in rodents [35]. The OF was a round plastic arena (d = 120 cm, h = 45 cm) with a central part of 60 cm in diameter; the OF was illuminated to 115 lx. Testing was performed 1 day before and on days 10, 20, and 30 after irradiation (Figure 1). The testing process was described in detail in our previous report [33]. Each animal was placed in the OF center and allowed to explore the arena for 5 min. During testing, mouse behavior was recorded with a WV-CP500G video camera (Panasonic, Osaka, Japan) and an EthoVision XT 8.5 video recording system (Noldus Information Technology, Netherlands); The videos were analyzed using EthoVision XT 8.5 software; the total distance traveled was measured and the time spent in the central zone was calculated and expressed as a percentage of the total time of the test. The number of rearings was determined.

### 2.3. Collection and Processing of Samples

In the peripheral blood, hemoglobin level and leukocyte count were determined. Blood was collected from the caudal vein at different terms of the experiment. In 3, 10, or 30 days after irradiation, the animals were anesthetized with isoflurane (Baxter, Baxter Healthcare Corporation of Puerto Rico, Deerfield, IL, USA) and decapitated; the organs (liver, spleen) and whole blood were collected.

#### 2.3.1. Measurement of Hemoglobin Level

The hemoglobin level was measured using an Easy Touch GCHb automatic analyzer (Bioptik Technology, Inc., Jhunan, Taiwan) before irradiation and on days 10, 20, and 30 after it (Figure 1). To this end, a drop of blood was applied to an Easy Touch hemoglobin test strip.

#### 2.3.2. Leukocyte Counting

Leukocytes were counted in the Goryaev chamber according to the standard procedure on days 10, 20, and 30 after irradiation (Figure 1). A blood sample (2.5 µL) was diluted by 20 times with 3% acetic acid with methylene blue.

#### 2.3.3. Differential Leukocyte Counting

For determining the percentage of different types of leukocytes, blood smears were prepared according to the standard method, stained by the Pappenheim method (successive staining with May-Grünwald and Romanovsky-Giemsa dyes), washed, dried, and examined under a Zeiss Imager Z2 VivaTome microscope (Carl Zeiss, Jena, Germany) with oil immersion. In each smear, 200 cells were counted and classified as neutrophils, lymphocytes, monocytes, eosinophils, or basophils based on morphological criteria.

#### 2.3.4. Calculation of the Absolute Number of Leukocytes of Different Types

Based on the total number of leukocytes and differential blood count, the absolute numbers of neutrophils, lymphocytes, monocytes, eosinophils, and basophils for each animal were calculated.

#### 2.3.5. Dynamic Light Scattering Analysis of Blood Serum (DLS)

Blood serum was obtained from mouse whole blood on days 3 or 30 after irradiation (Figure 1) The samples were left for 2 h at 4 °C, centrifuged at 3000 rpm for 10 min, and the supernatant was collected and stored at −20 °C until analysis. Changes in the subfraction composition of blood serum were evaluated using an LKS-03 laser correlation spectrometer (INTOX, St. Petersburg, Russia). To this end, the samples were thawed at room temperature, diluted 1:10 with saline, and transferred to a cuvette of the spectrometer in the volume of 0.2 mL. The method is based on the analysis of light scattering spectra obtained on particles present in biological fluids when a laser beam with coherent monochromatic radiation passes through the sample [36]. Processing of the obtained spectra yields size distribution histograms of particles present in the fluid and contributing to the light scatter.

### 2.4. Histological Analysis

The spleen and liver were isolated on days 3, 10, or 30 after irradiation and fixed in 4% neutral formalin (Figure 1). The preparations were processed routinely. The samples were embedded in paraffin, and tissue sections (thickness 5 µm) were sliced on a Leitz 1208 microtome (Leitz, Oberkochen, Germany). Dewaxed sections were routinely stained with hematoxylin and eosin by the Perls method [37] and by the van Gieson method (Van Gieson; ErgoProdaction LLC, St. Petersburg, Russia) according to manufacturer’s instructions. The stained sections were embedded in a mounting medium (DiaPath, Martinengo, Italy) and examined under a Zeiss Imager Z2 VivaTome light microscope (Carl Zeiss, Jena, Germany). At each of the above terms, organs of 7–10 mice in each group were examined.

### 2.5. Statistical Analysis

The statistical analysis was performed using GraphPad Prizm 8.0.1 software (La Jolla, San Diego, CA, USA). The normality of data distribution was assessed using the Shapiro–Wilk or Kolmogorov–Smirnov tests (depending on the sample sizes). In the case of normal distribution of the studied parameters, One-Way ANOVA was applied, followed by Tukey’s *post hoc* test or Šidák test for multiple comparisons. In cases where the hypothesis on the normal distribution of the test results cannot be accepted, a nonparametric one-way Kruskal–Wallis test was used for comparative analysis, followed by *post hoc* Dunn test for multiple comparisons. Animal survival was evaluated using the Kaplan–Meyer method (Gehan–Breslow–Wilcoxon test). The data are presented as the mean and standard error of the mean or as medians, quartiles, and minimum and maximum values. The differences were significant at *p* < 0.05.

## 3. Results

### 3.1. Effects of Lf on Survival Rate and Lifespan of Mice Exposed to Irradiation

**Figure 2:**
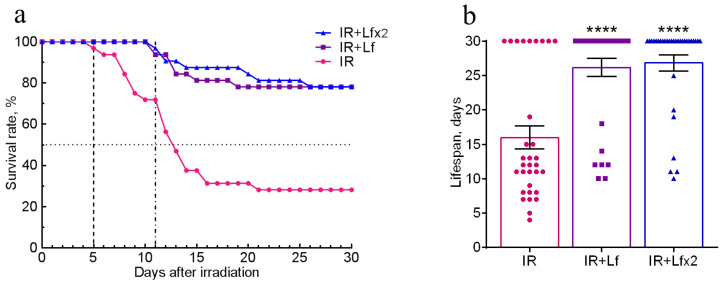
Effects of Lf on the survival rate (**a**) and lifespan (**b**) of mice after 7.5 Gy whole-body gamma irradiation. Lf (i.p.; 4 mg/mouse) was administered immediately after irradiation (IR+Lf and IR+Lf×2) and 24 h after it (IR+Lf×2). *n* = 32 in each group. The survival rates (%) and lifespan (days) during the 30-day period after irradiation are presented. **** *p* < 0.0001 in comparison with the IR group.

The effects of hLf on survival and mean lifespan (MLS) of mice (*n* = 32 in each experimental group) during the experiment were assessed daily over 30 days after irradiation. No animal deaths were recorded in the control groups throughout the experiment. In the IR group, the first deaths were recorded on day 5 and their number reached its maximum on days 7–14 after irradiation. By day 30, animal survival in this group was 28.1% (Figure 2a), and MLS was 16.0 ± 1.7 days (Figure 2b).

In the groups of IR+Lf and IR+Lf×2, the first death occurred on day 11 after irradiation (Figure 2a). It was found that administration of hLf increased survival rate to 78.1% in both groups of irradiated animals (Figure 2a), and MLS increased to 26.8 ± 1.2 (IR+Lf×2) and 26.2 ± 1.3 (IR+Lf) days (Figure 2b).

### 3.2. Effect of Lf on the Body Weight of Mice Exposed to Irradiation

**Figure 3:**
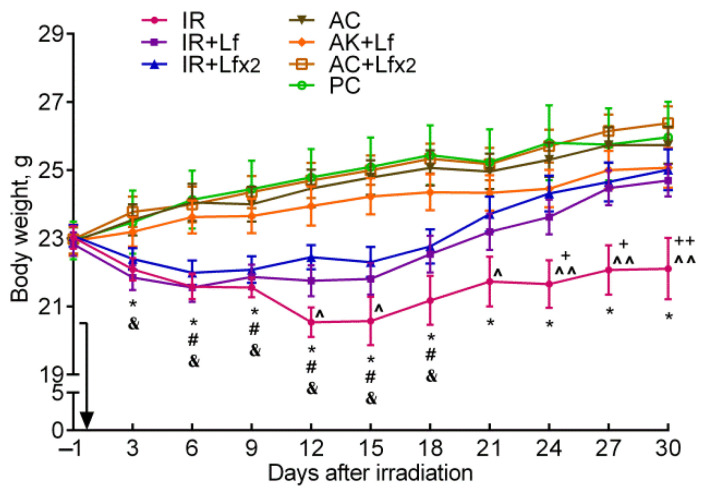
Effect of Lf on the body weight of mice after 7.5 Gy whole-body gamma irradiation. The arrow shows the day of irradiation. Lf (i.p.; 4 mg/mouse) was administered immediately after irradiation/sham-irradiation (IR+Lf, IR+Lf×2, AC+Lf, AC+Lf×2) and 24 h after it (IR+Lf×2, AC+Lf×2). *n* = 32 (for IR, IR+Lf, and IR+Lf×2); *n* = 17 (for AC, AC+Lf, and AC+Lf×2); *n* = 10 (PC). Values are presented as mean ± SEM. * *p* < 0.05—IR vs. AC; ^#^ *p* < 0.05—IR+Lf vs. AC+Lf; ^&^ *p* < 0.05—IR+Lf×2 vs. AC+Lf×2; ^+^ *p* < 0.05, ^++^ *p* < 0.01—IR vs. IR+Lf; ^ *p* < 0.05, ^^ *p* < 0.01—IR vs. IR+Lf×2 on the same day.

After irradiation, the body weight of the mice began to decrease, while in controls it gradually increased. In the control mice, hLf produced no appreciable effect on body weight gain; no differences by this parameter were found between the groups of active (AC, AC+Lf, AC+Lf×2) and passive control (PC) throughout the experiment. In all three experimental groups, a decrease in body weight was observed on day 3 after irradiation, although in the IR+Lf×2 group, this decrease was less pronounced (Figure 3).

In the experimental groups, the dynamics of body weight were different. In animals in the IR group, the body weight decreased until day 12, remained approximately constant on days 12–15 and 21–30, and increased on days 15–21. The body weight in irradiated animals treated with hLf decreased until day 6, remained practically constant on days 6–15, and then gradually increased.

In the IR group, the body weight was significantly lower than in the AC group starting from day 3 and until the end of the experiment; it was also lower than in the IR+Lf group on days 24–30 and in the IR+Lf×2 group on days 12, 15, and 21–30 (Figure 3). The animals in all experimental groups significantly differed by this parameter from the corresponding controls on days 6–18. By day 30, the body weight in the IR group mice did not return to the initial level. On the contrary, the body weight of animals in the IR+Lf and IR+Lf×2 groups reached the baseline level by day 21 and did not significantly differ from the corresponding controls.

### 3.3. Effects of Lf on Mouse Behavior in Open Field Test after Whole-Body Gamma Irradiation

The total distance traveled reflects motor activity [38], and the number of rearing is a measure of exploratory activity [39] of animals. All groups of mice showed similar motor activity and exploratory behavior before experimental exposures.

**Figure 4:**
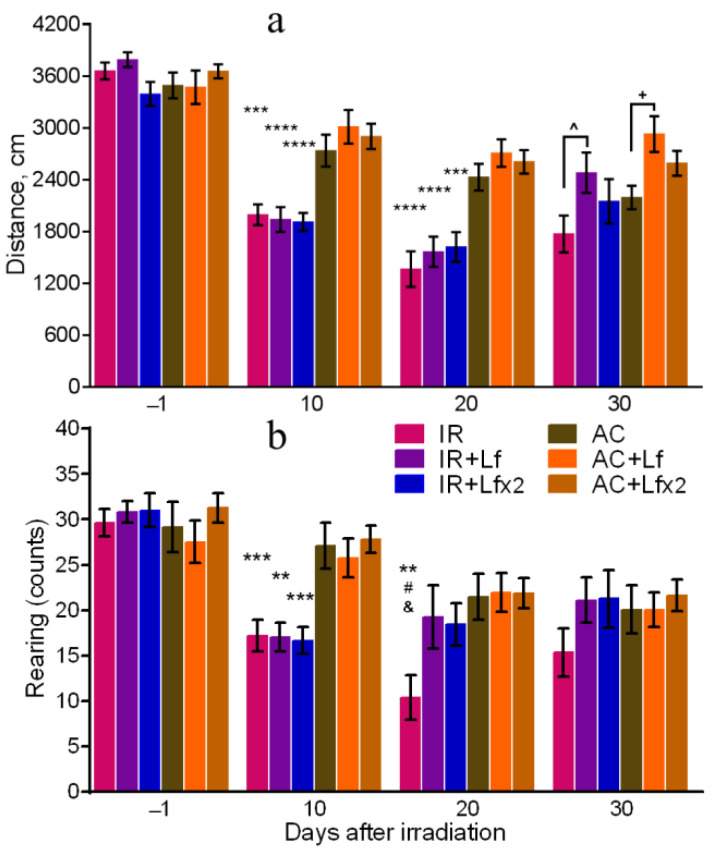
Effects of Lf on mouse behavior in the open field test after 7.5 Gy whole-body gamma irradiation. Lf (i.p.; 4 mg/mouse) was administered immediately after irradiation/sham-irradiation (IR+Lf, IR+Lf×2, AC+Lf, AC+Lf×2) and 24 h after it (IR+Lf×2, AC+Lf×2). Total distance traveled (**a**). Number of rearings (**b**). *n* = 32, 20, 20 (day −1; for IR, IR+Lf, and IR+Lf×2, respectively); *n* = 17 (for AC, AC+Lf, and AC+Lf×2). Values are presented as mean ± SEM. ** *p* < 0.01, *** *p* < 0.001, **** *p* < 0.0001 in comparison with the corresponding control (sham-irradiated) groups on the same day; ^#^ *p* < 0.05 in comparison with the IR+Lf group, ^&^ *p* < 0.05 in comparison with the IR+Lf×2 group on day 20. ^ *p* < 0.05; ^+^ *p* < 0.05.

On days 10 and 20 after irradiation, the total distance traveled by the mice in the OF in the experimental groups was lower than in the corresponding controls (Figure 4a). On day 30, the irradiated mice did not differ from the control animals by this parameter, but significant differences were revealed between the groups of IR and IR+Lf and between the groups AC and AC+Lf (Figure 4a). The mice that received a single injection of hLf passed a longer distance in the OF during the final testing.

The irradiated mice demonstrated a lower number of rearings on day 10 after the exposure in comparison with the control animals (Figure 4b). On day 20, this parameter in the IR group was significantly lower than in the AC (*p* < 0.01), IR+Lf (*p* < 0.05), and IR+Lf×2 (*p* < 0.05) groups, while experimental groups treated with hLf did not differ by this parameter from the corresponding controls. On day 30, there were no significant differences in the number of rearings between the groups.

**Figure 5:**
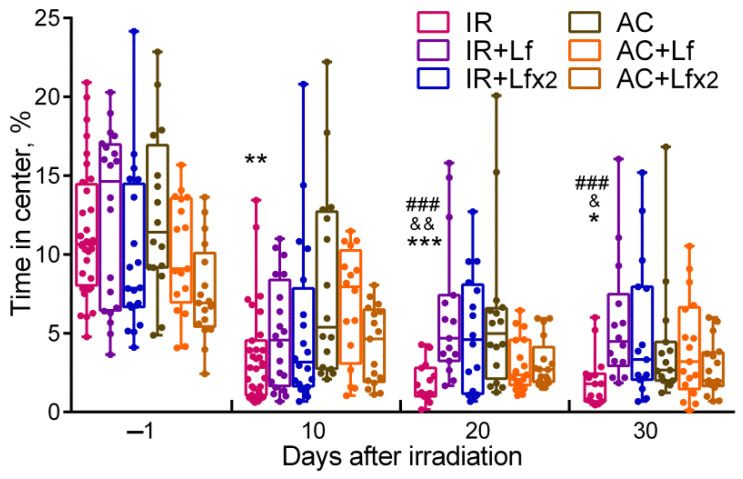
Effect of Lf on mouse behavior in the open field test after 7.5 Gy whole-body gamma irradiation. Lf (i.p.; 4 mg/mouse) was administered immediately after irradiation/sham-irradiation (IR+Lf, IR+Lf×2, AC+Lf, AC+Lf×2) and 24 h after it (IR+Lf×2, AC+Lf×2). Percent time spent in the center. Each dot represents a single animal. *n* = 32, 20, 20 (day −1; for IR, IR+Lf, and IR+Lf×2, respectively); *n* = 17 (for AC, AC+Lf, and AC+Lf×2). Data are presented as the median, quartiles, and min–max range. * *p* < 0.05, ** *p* < 0.01, *** *p* < 0.001 in comparison with the AC group; ^###^ *p* < 0.001 in comparison with the IR+Lf group; ^&^ *p* < 0.05; ^&&^ *p* < 0.01 in comparison with the IR+Lf×2 group on the same day.

By day 10, the mice in the IR group spent significantly less time in the central zone than the control animals (*p* < 0.01) (Figure 5), while on days 20 and 30, this parameter in the IR group was below the control (*p* < 0.001 and *p* < 0.05) and the corresponding values in groups IR+Lf (*p* < 0.001 and *p* < 0.001) and IR+Lf×2 (*p* < 0.01 and *p* < 0.05). By the time spent in the central zone, both experimental groups treated with hLf did not differ from the control groups at all stages of testing.

### 3.4. Effects of Lf on Changes in Blood Parameters of Mice Exposed to Irradiation

Blood samples were used to determine the following parameters: hemoglobin level (before and on days 3, 10, 20, and 30 after irradiation), total number of leukocytes (days 3, 10, and 30) and differential leukocyte count (days 3 and 30). The hematological parameters in the mice in the control groups (AC, AC+Lf, AC+Lf×2) did not differ from the reference values [24,40]. There were no differences in blood parameters between the active control groups (AC, AC+Lf, AC+Lf×2) during the experiment.

#### 3.4.1. Hemoglobin Level

**Figure 6:**
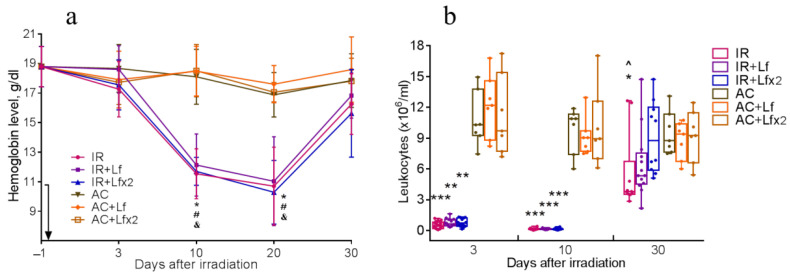
Dynamics of changes in the hemoglobin level (**a**) and the number of leukocytes (**b**) in the peripheral blood of mice after 7.5 Gy whole-body gamma irradiation. The arrow shows the day of irradiation. Lf (i.p.; 4 mg/mouse) was administered immediately after irradiation/sham-irradiation (IR+Lf, IR+Lf×2, AC+Lf, AC+Lf×2) and 24 h after it (IR+Lf×2, AC+Lf×2). (**a**) *n* = 21, 15, 15 (day −1; for IR, IR+Lf, and IR+Lf×2, respectively); *n* = 9 (for AC, AC+Lf, and AC+Lf×2). * *p* < 0.05—IR vs. AC; ^#^ *p* < 0.05—IR+Lf vs. AC+Lf; ^&^ *p* < 0.05—IR+Lf×2 vs. AC+Lf×2 on the same day. (**b**) Each dot represents a single animal. *n* = 20, 15, 12 (day −1; for IR, IR+Lf, and IR+Lf×2, respectively); *n* = 7 (for AC, AC+Lf, and AC+Lf×2). * *p* < 0.05, ** *p* < 0.01, *** *p* < 0.001 in comparison with the corresponding control (sham-irradiated) groups on the same day; ^ *p* < 0.05 in comparison with group IR+Lf×2 on day 30.

Irradiation caused a significant decrease in hemoglobin levels. On days 10 and 20, mice of all experimental groups differed by this parameter from the corresponding controls (Figure 6a). By day 30, the hemoglobin concentration in irradiated animals almost returned to the initial levels.

#### 3.4.2. Total Leukocyte Count

Irradiation caused a sharp decrease in the leukocyte count in all experimental groups. On days 3 and 10, the groups IR, IR+Lf, and IR+Lf×2 significantly differed from the corresponding controls by this parameter (Figure 6b).

On day 30, the leukocyte count in the experimental groups increased, while in the group IR it remained significantly lower than control (*p* < 0.05). On day 30 after irradiation, the mice in groups IR+Lf and IR+Lf×2 did not differ from the corresponding controls by this parameter. In addition, the number of leukocytes in the IR+Lf×2 group on day 30 was significantly higher than in the IR group (*p* < 0.05).

#### 3.4.3. Differential Leukocyte Count

**Figure 7:**
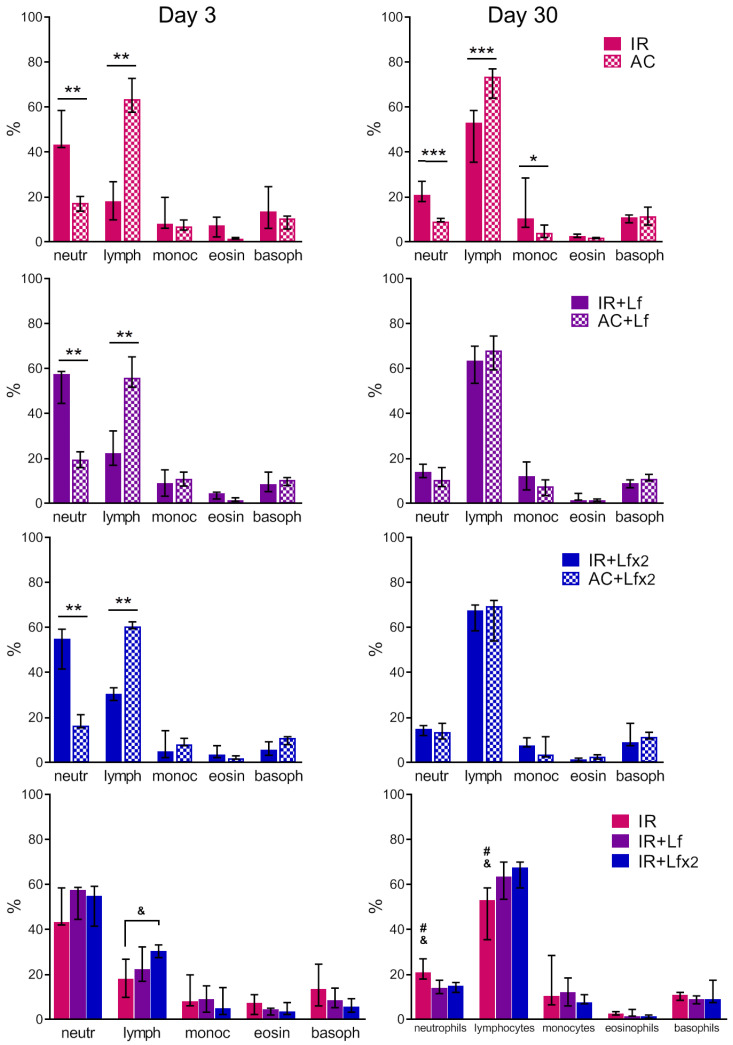
Effects of Lf on differential leukocyte count in the mice on days 3 (left) and 30 (right) after 7.5 Gy whole-body gamma irradiation. The relative content of blood cells (%). *n* = 5–6 (day 3), *n* = 7 (day 30) for each group. Data are presented as median ± interquartile range. * *p* < 0.05, ** *p* < 0.01, *** *p* < 0.001; ^#^ *p* < 0.05 in comparison with the IR+Lf group; ^&^ *p* < 0.05 in comparison with the IR+Lf×2 group.

In the experimental groups, depletion of the lymphocyte reserve and an increase in the relative number of neutrophils were observed on day 3 after irradiation (Figure 7, left). The relative number of leukocytes in the IR+Lf×2 group was significantly higher than in the IR group (*p* < 0.05). The number of monocytes, eosinophils, and basophils in the irradiated mice did not differ from the control values.

In both experimental groups treated with hLf, the differential leukocyte count was restored by day 30 (Figure 7, right). The relative number of monocytes in the animals in the IR group on day 30 was higher than in the control (*p* < 0.05). In the IR group, a trend to normalization of the differential leukocyte count was observed, though the relative numbers of lymphocytes and neutrophils in this group significantly differed from the corresponding parameters in groups AC (*p* < 0.001), IR+Lf (*p* < 0.05) and IR+Lf×2 (*p* < 0.05) on day 30 after irradiation.

The absolute numbers of leukocytes of different types in the blood of mice on days 3 and 30 after irradiation are shown in Table 1 and Table 2, respectively. A dose-dependent effect of hLf on the recovery of the lymphocyte and basophil number was revealed on day 30.

### 3.5. Effects of Lf on Changes in Subfraction Composition of the Blood Serum after Gamma Irradiation

The dynamic light scattering (DLS) method based on the measurement of the spectral characteristics of light scattering allows for assessing particle size in biological fluids and their ratio [41]. The results of measurement are usually presented in the form of a histogram, where the abscissa corresponds to the size of particles (in nm) and the ordinate shows their contribution to the light scatter (in %).

**Figure 8:**
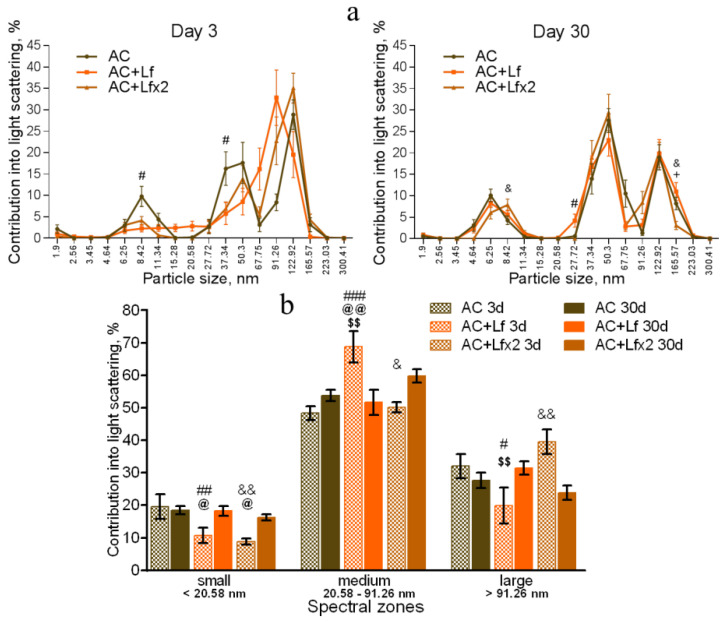
Changes in the DLS spectra of the blood serum from mice in the control groups on days 3 and 30 after sham-irradiation. *n* = 5–6 (day 3), *n* = 12–14 (day 30) for each group. Particle size distribution (**a**). ^#^ *p* < 0.05—AC+Lf vs. AC, ^&^ *p* < 0.05—AC+Lf×2 vs. AC, + *p* < 0.05—AC+Lf vs. AC+Lf×2. Particle distribution by spectral zones (**b**). ^#^ *p* < 0.05, ^##^ *p* < 0.01, ^###^ *p* < 0.001, ^&^ *p* < 0.05, ^&&^ *p* < 0.01 in comparison with the same group on day 30; ^@^ *p* < 0.05, ^@@^ *p* < 0.01 in comparison with the AC group on day 3; ^$$^ *p* < 0.01 in comparison with the AC+Lf×2 group on day 3.

**Figure 9:**
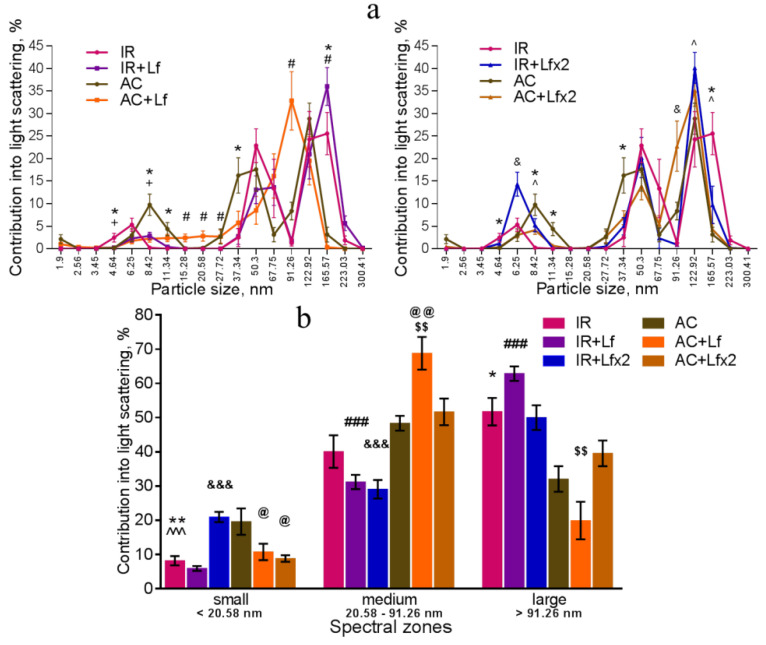
Changes in the DLS spectra of mouse blood serum on day 3 after 7.5 Gy whole-body gamma irradiation. Particle size distribution (**a**). Particles distribution by spectral zones (**b**). *n* = 5–6 for each group. * *p* < 0.05, ** *p* < 0.01—IR vs. AC, ^#^ *p* < 0.05, ^###^ *p* < 0.001—IR+Lf vs. AC+Lf, ^&^ *p* < 0.05, ^&&&^ *p* < 0.001—IR+Lf×2 vs. AC+Lf×2, ^+^ *p* < 0.05—IR vs. IR+Lf, ^ *p* < 0.05, ^^^ *p* < 0.001—IR vs. IR+Lf×2, ^@^ *p* < 0.05, ^@@^ *p* < 0.01 in comparison with the AC group; ^$$^ *p* < 0.01 in comparison with the AC+Lf×2 group.

**Figure 10:**
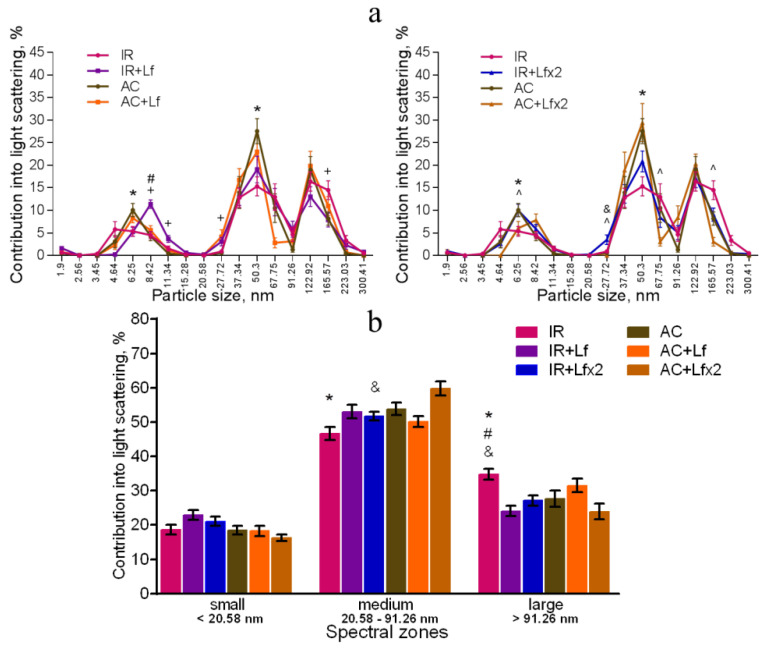
Effect of Lf on the changes in the DLS spectra of mouse blood serum on day 30 after 7.5 Gy gamma irradiation. Lf (i.p.; 4 mg/mouse) was administered immediately after irradiation/sham-irradiation (IR+Lf, IR+Lf×2, AC+Lf, AC+Lf×2) and 24 h after it (IR+Lf×2, AC+Lf×2). Particle size distribution (**a**). Particles distribution by spectral zones (**b**). *n* = 9–15 for each group. * *p* < 0.05—IR vs. AC, ^#^ *p* < 0.05—IR+Lf vs. AC+Lf, ^&^ *p* < 0.05—IR+Lf×2 vs. AC+Lf×2, ^+^ *p* < 0.05—IR vs. IR+Lf, ^ *p* < 0.05—IR vs. IR+Lf×2.

In our experiment, the DLS spectra of mouse serum had a form of a three-modal distribution (Figure 8a, Figure 9a and Figure 10a). In these DLS spectra, three discrete zones were distinguished corresponding to small (<20.58 nm), medium (20.58–91.26 nm), and large (>91.26 nm) particles [30]; and the contribution of particles in each of these zones to the light scatter was estimated.

The DLS spectra of the serum from AC group mice remained constant throughout the experiment. On day 30, the distribution of serum particles did not differ from that on day 3 (Figure 8a,b). Single and repeated injections of hLf to the control animals (groups AC+Lf and AC+Lf×2) led to a significant decrease in the contribution of small particles to the light scatter on day 3 (Figure 8b); on day 30, this parameter returned to the level of the AC group.

In groups AC+Lf and AC+Lf×2, different dynamics of the distribution of medium and large particles was observed. A single injection of hLf increased the contribution of medium particles (67.75–91.26 nm) and decreased the contribution of large particles (122.92–165.57 nm) on day 3. In the AC+Lf group, only one peak corresponding to the large-molecular fraction was seen; the position of this peak was shifted towards medium particles (Figure 8a). The distribution of serum particles in this group was restored by day 30.

Repeated injections of hLf changed the effect of the first dose observed on day 3: contributions of medium and large particles in the group AC+Lf×2 did not differ from those in the AC group. On day 30, the contribution of large particles to the light scatter slightly decreased, and the contribution of medium particles increased, while both indicators did not differ from the corresponding indicators in the AC group. Thus, the DLS spectra of the serum in the control groups did not significantly differ from each other in all three spectral zones on day 30. In all three control groups, the maximum contribution to the light scatter on days 3 and 30 was made by medium particles (Figure 8b).

Irradiation induced significant changes in the DLS spectra. A common pattern of changes in the subfraction composition of the serum after irradiation was an increase in the contribution of large particles (>122.92 nm) [42]. This reaction was observed in all experimental groups on day 3 after irradiation (Figure 9a,b).

In the irradiated animals, a single injection of hLf was followed by an increase in the contribution of large particles (165.57–223.03 nm) and a decrease in the contribution of medium particles to the light scatter (Figure 9a left, b). Repeated injections of hLf led to a slight increase in the contribution of particles with a size of 122.92 nm and a significant increase in the contribution of small particles (6.25–8.42 nm) (Figure 9a right, b). In the sera of irradiated mice (groups IR, IR+Lf, IR+Lf×2) on day 3, the maximum contribution to the light scatter was made by large particles. Repeated injections of hLf (group IR+Lf×2) led to slight shifts towards the control DLS histograms, and a predominant peak corresponding to large particles (122.92 nm) appeared. The position of this peak and the contribution to the light scatter corresponded to the values in the control groups (Figure 9a right).

On day 30, the contribution of medium particles to the light scatter was significantly lower, and the contribution of large particles remained significantly higher in the IR group in comparison with the control (Figure 10a,b). In addition, the contribution of large particles in irradiated animals (IR group) was higher than in mice treated with hLf after irradiation (groups IR+Lf and IR+Lf×2). The position of the peak corresponding to small particles in the IR group was shifted to the left (4.64 nm) in comparison with the control (6.25 nm), and the contribution of medium particles (50.3 nm) to the light scatter in this experimental group was significantly lower than in the AC group (Figure 10a). Thus, the subfraction distribution of serum particles in irradiated animals (IR group) did not return to normal by the end of the experiment, which is consistent with previously published data [42].

On day 30, the DLS spectra of mouse sera in groups IR+Lf and IR+Lf×2 practically did not differ from those in the control groups (AC, AC+Lf, AC+Lf×2) in all three spectral zones by the position of peaks and their percentage contribution to the light scatter. At the same time, a decrease in the contribution of medium particles to the light scatter was found in the IR+Lf×2 group in comparison with the AC+Lf×2 group. The position of the small particle peak was slightly shifted towards medium particles (8.42 nm) in groups IR+Lf (Figure 10a left) and AC+Lf×2 (Figure 10a right).

### 3.6. Histological Analysis of Mouse Spleen and Liver after Total Gamma Irradiation

Histological analysis of the spleen and liver was performed on days 3, 10, and 30 after irradiation. It is known that acute exposure to high doses of ionizing radiation causes significant morphological changes in these organs [43,44].

#### 3.6.1. Spleen

**Figure 11:**
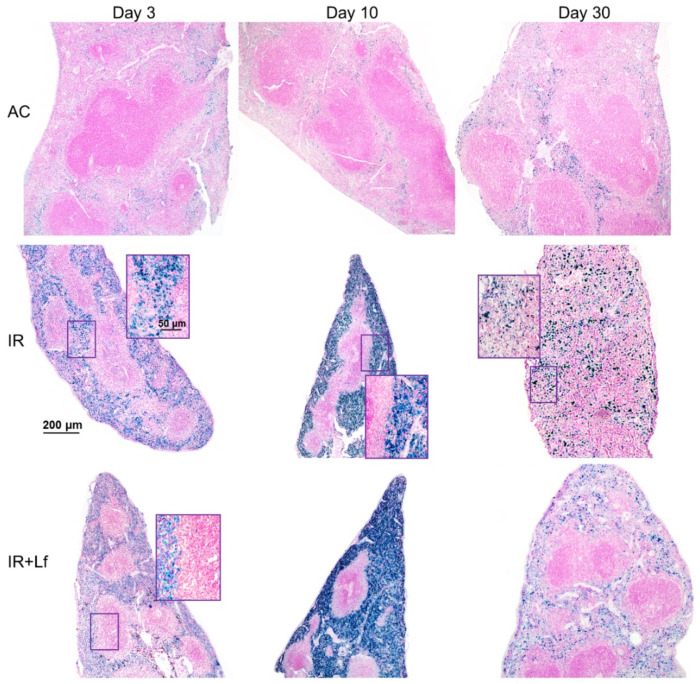
Protective effect of Lf on the structural organization of the spleen in the irradiated mice. Representative photomicrographs of spleen sections on days 3 (left column; *n* = 5–6 for each group), 10 (middle column; *n* = 5–6 for each group), and 30 (right column; *n* = 7–10 for each group) days after 7.5 Gy whole-body gamma irradiation. Groups: AC (sham-irradiated), IR (irradiated), IR+Lf (Lf was administered immediately after irradiation; i.p.; 4 mg/mouse). Pearl’s staining for iron (III). Scale bars = 200 μm and 50 μm.

**Figure 12:**
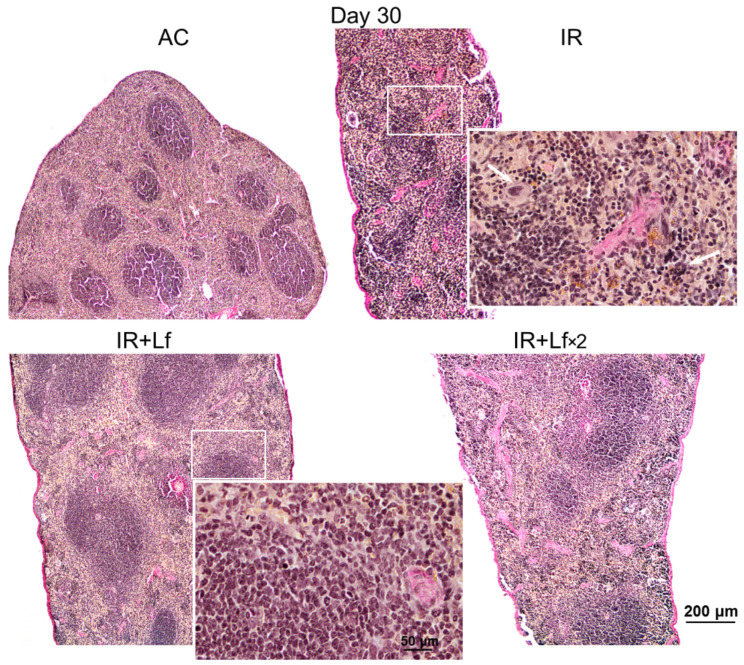
Protective effect of Lf on the structural organization of the spleen in the irradiated mice. Representative photomicrographs of spleen sections on day 30 after 7.5 Gy whole-body gamma irradiation. Groups: AC (sham-irradiated), IR (irradiated), IR+Lf (Lf was administered immediately after irradiation; i.p.; 4 mg/mouse), IR+Lf×2 (Lf was administered twice: immediately after irradiation and 24 h after it). Megakaryocytes (arrows). van Gieson staining. Scale bars = 200 μm and 50 μm.

In animals in the control groups (AC, AC+Lf, AC+Lf×2), the spleen had smooth clear-cut contours at all studied terms (days 3, 10, and 30) (Figure 11 and Figure 12). The main structural elements (white and red pulp, trabeculae) were clearly distinguished on sections. The lymphoid follicles in the white pulp were of medium size with clear boundaries and lighter germinal centers located in the central part. The vascular walls were not changed, moderate blood filling of the red pulp was observed. A small amount of hemosiderin was found in macrophages in the red pulp. The administration of hLf (groups AC+Lf, AC+Lf×2) did not induce histological changes in the organ.

Irradiation led to the accumulation of hemosiderin in the red pulp of the spleen in mice of all experimental groups (IR, IR+Lf, IR+Lf×2); the content of hemosiderin increased significantly from day 3 to day 10 and then decreased but remained above the control level on day 30 (Figure 11).

In mice in the group IR, the ratio of the white and red pulp changed as soon as on day 3 after irradiation; on day 10, reduction in follicles began, the boundaries of follicles disappeared (Figure 11). On day 30, the spleens of the IR group animals had no clear division in the white and red pulp, the follicles were reduced. Solitary giant cells of irregular shape morphologically similar to megakaryocytes were somewhere seen (Figure 12). The decrease in the total volume of the organ led to the appearance of folds on its surface. In both experimental groups, IR+Lf and IR+Lf×2, the structural organization of the spleen was preserved throughout the experiment (Figure 11 and Figure 12).

#### 3.6.2. Liver

In animals in the control groups (AC, AC+Lf, AC+Lf×2), the liver tissue had a cord structure, the cells had clear-cut boundaries at all terms of the experiment (days 3, 10, and 30). Hepatocytes had a polygonal shape, granular cytoplasm, and contained one or sometimes two round or elongated nuclei with clear-cut contours and chromatin lumps. The administration of hLf (groups AC+Lf, AC+Lf×2) did not change the liver structure.

**Figure 13:**
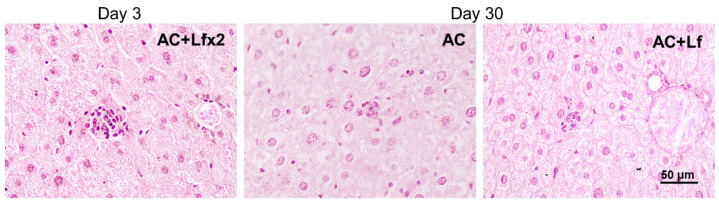
Solitary clusters of mononuclear cells in the liver parenchyma of control mice. Representative photomicrographs of liver sections on days 3 (left) and 30 (middle; right) after sham-irradiation. Groups: AC, AC+Lf, AC+Lf×2. Hematoxylin and Eosin staining. Scale bar = 50 μm.

Solitary clusters of mononuclear cells were observed in the liver parenchyma of the control mice (AC group). In the animals in groups AC+Lf and AC+Lf×2, these clusters were larger and more numerous as soon as day 3 (Figure 13 left); the same picture was also observed on day 10. On day 30, clusters of mononuclear cells in the animals of all the control groups (AC, AC+Lf, AC+Lf×2) had similar size (Figure 13 middle, right), but their number was higher in group AC+Lf×2.

**Figure 14:**
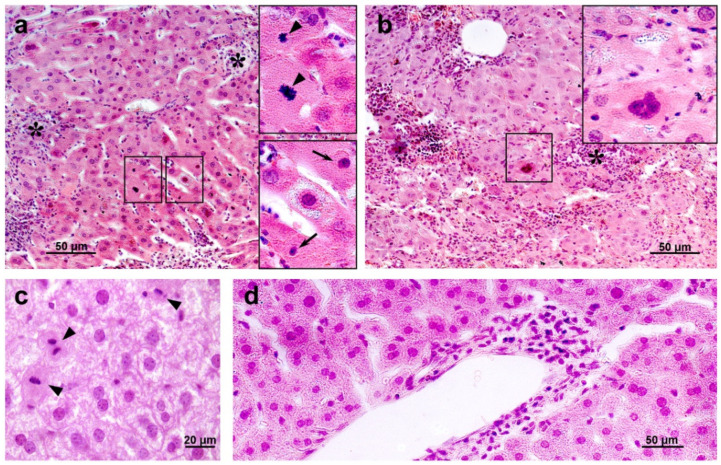
The histological changes in mouse liver after 7.5 Gy whole-body gamma irradiation: necrosis ((**a**,**b**), asterisks), pyknotic nuclei ((**a**), arrows), numerous mitoses ((**a**,**c**), arrow heads), vacuolar dystrophy of hepatocytes (**c**), mononuclear infiltration (**d**). Megakaryocyte (**b**). Representative photomicrographs of liver sections (groups: IR, IR+Lf, IR+Lf×2). Hematoxylin and Eosin staining. Scale bars = 50 μm and 20 μm.

Histological examination revealed degenerative changes of varying severity in the liver tissue of the IR group mice; they appeared as soon as day 3 after irradiation and persisted on days 10 and 30. Numerous abnormalities in the hepatocyte nuclei were revealed: vacuolization, pyknosis, chromatin condensation; appearance of trinuclear and an increase in the number of binuclear cells; vacuolar degeneration (Figure 14). The sinusoids were dilated, the central veins had increased diameter, the interlobular connective tissue was poorly developed. We also observed focal necrosis of hepatocytes and multiple mononuclear infiltrates in the periportal zone that developed against the background of circulatory disorders. The number of mitoses in hepatocytes was increased, spindle disturbances were somewhere seen. The maximum number of mitoses was observed on day 10 of the experiment. In the liver tissue, giant cells of irregular shape morphologically similar to megakaryocytes were somewhere seen (Figure 14b). The administration of hLf to the irradiated animals (groups IR+Lf and IR+Lf×2) had no effect on the severity of the above-mentioned reactive changes on days 3, 10, and 30.

**Figure 15:**
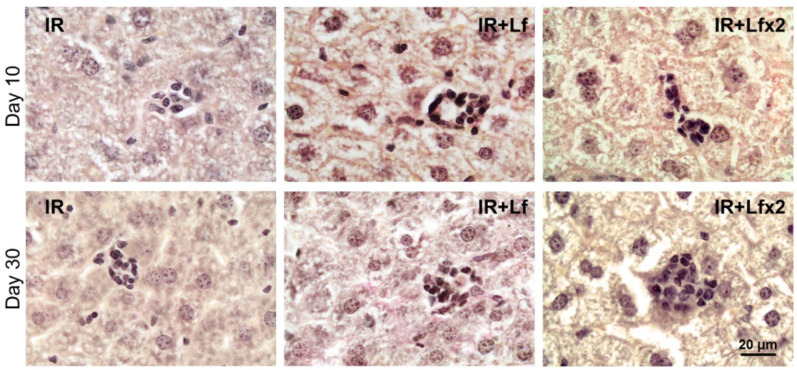
Lf promoted recovery of mononuclear cell clusters in the liver parenchyma of mice exposed to 7.5 Gy whole-body gamma irradiation. Representative photomicrographs of liver sections on days 10 (upper; *n* = 5–6 for each group) and 30 (bottom; *n* = 7–10 for each group) after irradiation. Groups: IR (irradiated), IR+Lf (Lf was administered immediately after irradiation; i.p.; 4 mg/mouse), IR+Lf×2 (Lf was administered twice: immediately after irradiation and 24 h after it). van Gieson staining. Scale bar = 20 μm.

Irradiation led to the disappearance of clusters of mononuclear cells in the liver parenchyma in animals of all the experimental groups (IR, IR+Lf, IR+Lf×2) on day 3 after the exposure. These structures started to recover on day 10 of the experiment (Figure 15 upper). By day 30, the number and size of these clusters were significantly higher in the irradiated mice treated with hLf (IR+Lf, IR+Lf×2) than in the IR group animals (Figure 15 bottom) and close to the corresponding control values (groups AC+Lf, AC+Lf×2, respectively).

## 4. Discussion

The exposure to high doses of ionizing radiation can induce significant body weight loss and lead to animal death. For instance, the body weight of mice exposed to whole-body gamma irradiation in a dose of 8 Gy decreased by 15% on day 14 [31], in a dose of 7.5 Gy—on day 9 [45]. It was shown that the mouse survival rate on day 30 was 20% [31,46] or 13% [47] after whole-body gamma irradiation in a dose of 8 Gy and 33% after X-ray irradiation in a dose of 7 Gy [24], while the first deaths of animals were recorded on days 9, 5, 10, or 6, respectively.

In our study, a similar moderate body weight loss was observed in all experimental groups within the first 6 days after irradiation. In the IR group, the first wave of intensive animal deaths was observed on day 5; by day 9, 75% of mice remained alive (Figure 2a). A sharp decrease in mouse body weight in the IR group on days 9–12 was followed by a second wave of deaths on days 12–16 (survival rate 31%); after that, some more animals died. By day 30, the body weight in the IR group mice did not return to the initial level. Injections of hLf made it possible to prolong the lifespan of irradiated mice. The first death in both experimental groups treated with hLf occurred only on day 11. The weight loss in these groups continued up to day 6 and then the animals progressively gained weight from day 15 to the end of the experiment. By day 21, the body weight of animals in groups IR+Lf and IR+Lf×2 returned to the initial level and did not differ from the corresponding controls (Figure 3). It was shown that single and repeated administration of hLf increased the survival rate of irradiated mice from 28 to 78% during the experiment (Figure 2a) and had a compensatory effect on body weight loss in irradiated animals.

These findings agree with previous reports. On day 30 after whole-body X-ray irradiation in a dose of 6.8 Gy or 7 Gy, the survival rate of mice receiving a diet containing 0.1% bovine Lf for 7 days before and 30 days after irradiation was higher: 85 compared to 62% [4] or 50 compared to 33% [24], respectively. Bovine Lf intraperitoneally injected in mice (4 mg/animal) immediately after X-ray irradiation in a dose of 6.8 Gy increased the 30-day survival rate from 50 to 92% [4]. In guinea pigs subjected to whole-body gamma irradiation in a dose of 2.5 Gy, daily subcutaneous injections of Lf obtained by a biotechnological method from rabbit colostrum in doses of 65 or 300 µg/kg on days 1–14 after the exposure increased the 30-day survival rate from 54 to 86% or 100%, respectively [48]. It was reported that after X-ray irradiation in a dose of 7 Gy, the body weight of mice decreased until day 10 and then began to increase; in animals irradiated against the background of a diet containing 0.1% bovine Lf (7 days before and 30 days after), this indicator on days 20–30 was significantly higher [24]. One of the main causes of body weight loss in irradiated animals is radiation damage to the intestinal mucosa. In mice subjected to X-ray irradiation in a dose of 5 Gy, intraperitoneal injections of bovine Lf (2 or 4 mg/animal, 4 h before and once a day over 3 days after irradiation) alleviated damage to the small intestinal epithelium (assessed by the length of the intestinal villi and the ratio of villus length to crypt depth) [25].

X-ray irradiation in a dose of 10 Gy led to a decrease in motor activity and aggressive behavior in mice [49]. The suppression of motor and exploratory activity was observed in mice after gamma irradiation in a dose of 8 Gy, slow recovery of these parameters began only after 17 days [46]. Acute X-ray irradiation in a dose of 6 Gy reduced motor activity and coordination of movements and increased anxiety tested 72 h after the exposure [50].

Our findings obtained in behavioral experiments for the IR group are consistent with the results of the reports cited above. The administration of hLf had a compensatory effect on the radiation-induced decrease in the exploratory activity of mice (Figure 4b) and prevented a change in their behavior in terms of the time spent in the OF center (Figure 5). These data were obtained by us for the first time.

Changes in the composition of peripheral blood are one of the main criteria for assessing the effects of radiation on the body. Previous studies showed that mouse survival after sublethal irradiation depends on recovery of the hematopoiesis system [31,51]. It is also known that the decrease in leukocyte count correlates with the radiation dose [52].

Our experiments showed that irradiation caused a significant decrease in the hemoglobin level (Figure 6a) and leukocyte count (Figure 6b); the latter parameter did not return to normal by day 30. These findings are consistent with the reports of other researchers. After X-ray irradiation in doses of 6.8 and 7 Gy, the hemoglobin level in mice was significantly reduced on day 15 [4] and between days 7 and 29 [24], and after gamma irradiation in doses of 5 Gy—between days 7 and 14 [53]. The number of leukocytes in the peripheral blood decreased significantly 3 days after whole-body gamma irradiation in a dose of 8 Gy and did not return to the normal range within 21 days [31]. After whole-body X-ray irradiation in a dose of 7 Gy, the leukocyte count rapidly decreased until day 14, then began to increase gradually but remained significantly below the control level on day 29 [24]. In our experiments, the animals of the experimental groups treated with hLf did not differ from the corresponding control groups by this parameter on day 30 after irradiation (Figure 6b). This is in line with the study of Feng et al., in which the number of peripheral blood leukocytes in animals treated with bovine Lf over 7 days before irradiation (7 Gy) and 30 days after it returned to normal by day 29 [24].

Each type of blood cell has its own relative radiosensitivity; and it is highest in lymphocytes [54]. The exposure to ionizing radiation in a high dose leads to the depletion in lymphocyte stores and an increase in the relative number of neutrophils [54]. This particular reaction was observed in all experimental groups on day 3 after irradiation (Figure 7, left; Table 1). Neither relative nor absolute numbers of different types of leukocytes in animals in the IR group recovered by day 30 (Figure 7, right, Table 2). This can indicate that a sufficient renewal of leukocytes after radiation damage was not achieved by this term. It was previously shown that the blood content of lymphocytes and neutrophils in mice was not restored on day 30 after whole-body gamma irradiation in a dose of 8 Gy [47].

In both experimental groups treated with hLf, the differential leukocyte count was restored by day 30 (Figure 13, right). Moreover, a dose-dependent effect of hLf on the recovery of the absolute number of lymphocytes and basophils was revealed on day 30 (Table 2). This is in line with previously published reports. It was shown that long-term administration of Lf (65 or 300 µg/kg, subcutaneously, daily from day 1 to day 14) obtained from rabbit colostrum promoted normalization of the cellular composition of the peripheral blood in guinea pigs after deep suppression of hematopoiesis induced by gamma irradiation in a dose of 2.5 Gy and increased the content of lymphocytes (day 12) and neutrophils (days 16 and 18) [48]. The data presented by us suggest that hLf had a compensatory effect on the radiation-induced decrease in the total number of leukocytes in the blood and changes in the differential leukocyte count in mice.

Irradiation induced significant shifts in the subfraction distribution of serum particles in mice (Figure 9), which is consistent with previously published data [42]. In our DLS study, we showed for the first time that administration of hLf promoted recovery of serum homeostasis parameters to their normal values by the end of the experiment (Figure 10).

It is known that acute exposure to high doses of ionizing radiation induces significant morphological changes in various organs [43,44]. In 3 and 6 weeks after single whole-body gamma irradiation in a dose of 7.5 Gy, fatty degeneration of hepatocytes, intensive fibrosis, increased number of cells with mitosis figures, increased number of apoptotic cells and cells with nuclear abnormalities, dilation of sinus vessels, focal necrosis of hepatocytes, and multiple mononuclear infiltrates in the liver parenchyma were observed [30,43]. In the spleen, the absence of clear-cut subdivision of the parenchyma into the white and red pulp, the absence of boundaries between the follicles, increased content of macrophages and leukocytes, and solitary megakaryocytes were observed in the same studies. The data obtained by us are consistent with these reports (Figure 11, Figure 12 and Figure 14). It should be noted that hLf had a protective effect on the structural organization of the spleen in irradiated animals (Figure 11 and Figure 12).

In our study, hLf produced pleiotropic effects on mice subjected to gamma irradiation, which indicates that it affects several targets triggering various biochemical processes in the body of experimental animals. The mechanism underlying the effects of Lf in irradiated animals remains poorly understood. The positive effect of Lf can be related to its antioxidant properties [4,5]. The development of infectious diseases due to impaired immunity is a cause of postirradiation death. The radioprotective effect of Lf can be related to its immunomodulatory function [9]. It was shown that exogenous Lf suppresses the expression of some pro-inflammatory cytokines (tumor necrosis factor and interleukins −1β and −6) and activates the expression of anti-inflammatory factors (IL-4 and IL-10) [11,25]. In our study, hLf had a protective effect on the structural organization of the spleen, an immune system organ. It was previously shown that Lf alleviated damage to the small intestinal epithelium [25] and increased survival by stimulating recovery of the intestinal microflora and inhibiting the development and exacerbation of infectious diseases [4] in mice subjected to irradiation.

The degree of damage to the hematopoietic function positively correlates with the radiation dose [31]. The bone marrow, the primary hematopoietic tissue, is highly sensitive to irradiation. Even a low dose of ionizing radiation can disrupt the hematopoietic balance in the bone marrow, while the resulting anemia, bleeding, and infections have a serious impact on animal survival. Hematopoiesis is an intricate process regulated by numerous factors. Even though the bone marrow is the main organ of hematopoiesis, it can occur in many other tissues both during intrauterine development and after birth. In mice, the spleen remains a hematopoietic organ throughout their lives, although the intensity of splenic hematopoiesis is low. During adult life, the liver maintains hematopoietic stem cells, erythropoiesis and myelopoiesis also at a low level [55,56]. After birth, extramedullary hematopoiesis is observed in mammals (in particular, in rodents) when immune reactions occur at the periphery, as a normal reaction to infection and inflammation, as a response to bleeding, hemolysis, and radiation exposure. The liver and the spleen are the main organs that can become the sites of extramedullary hematopoiesis [57].

In contrast to rapid activation of erythropoiesis in the spleen in response to peripheral anemic stress, erythropoiesis in this organ after acute radiation stress can be restored only after recovery of the bone marrow. Within 6 days after acute whole-body gamma irradiation in a dose of 4 Gy, the basal level of erythropoiesis in the mouse spleen was absent despite rapid recovery of the bone marrow, while within 10 days, significant enlargement of the red pulp was noted and histological analysis confirmed extramedullary erythropoiesis in the spleen [58]. Extensive extramedullary hematopoiesis was observed in the mouse spleen on day 10 after gamma irradiation in a dose of 6 Gy [59]. In our experiment, the mice were exposed to whole-body gamma radiation in a higher dose (7.5 Gy). This can explain the increase in the number of leukocytes in the blood of animals in the experimental groups only on day 30 but not on day 10, as in the above-mentioned studies. To clarify the dynamics of recovery of animals by this parameter, the blood samples should be examined at intermediate terms between days 10 and 30 after irradiation. On day 30, megakaryocytes were identified in histological sections of the spleen (IR group) and liver (IR, IR+Lf, and IR+Lf×2 groups), which can be indicative of active extramedullary hematopoiesis in these organs [60]. It can also be assumed that solitary clusters of mononuclear cells in the liver parenchyma (Figure 15) represented the foci of extramedullary hematopoiesis that disappeared on day 3 and started to recover on day 10 after irradiation. Administration of hLf led to an increase in the number and size of these clusters.

In the normal liver, constantly changing metabolic and tissue remodeling activity combined with regular exposure to microbial products results in persistent regulated inflammation [61]. Activation of inflammatory processes is closely related to mechanisms that eliminate inflammation and promote tissue regeneration. In our experiments, the postirradiation death of lymphocytes led to the suppression of local immunity in the liver and the disappearance of mononuclear cell clusters in the liver parenchyma. The treatment with hLf stimulated local immunity by attracting immunocompetent cells to the liver tissue when their number in the circulation increased. It can be assumed that these clusters in the liver parenchyma consisted of immunocompetent cells.

The regenerative potential of the liver is determined by inflammatory mediators (e.g., IL-1α, TNFα, IL-6), growth factors (e.g., hepatocyte growth factor), and populations of immune cells located in the liver [62]. Kupffer cells play a central role in this regeneration through the release of IL-6 and TNFα, which promotes hepatocyte proliferation, while depletion of Kupffer cells prevents subsequent liver regeneration [63]. It is known that Lf can bind to macrophages and activate these cells [64,65]. In addition, in a mouse model of liver damage induced by acetaminophen (300 mg/kg, intraperitoneally), which directly affects hepatocytes by inducing mitochondrial dysfunction, lipid peroxidation, oxidative stress, and DNA fragmentation, bovine Lf (50 mg/kg, intravenously) administered 1 and 4 h after acetaminophen injection prevented liver damage in animals [66]. Lf exhibited its hepatoprotective effect only in the presence of Kupffer cells. Researchers believe that Lf protects hepatocytes by stimulating production of protective mediators by Kupffer cells.

As noted above, Lf can perform many different functions depending on the cellular system it affects, due to the specificity of the receptors. It is known that exogenous Lf is mainly utilized in the liver [67,68]. Here, it can directly affect hepatocytes, endothelial cells, and Kupffer cells that express receptors to this protein (LRP1 and asialoglycoprotein receptor, intelectin-1, and CD14, respectively).

Of great interest is the ability of Lf to modulate a whole range of processes by changing the expression of genes encoding some regulatory and effector proteins [69,70]. In our previous studies, we found that in neuronal cultures under conditions of stimulation, Lf enhances the expression of transcription factor c-Fos, a marker of neuronal activity and long-term plasticity [71]. Lf binding to the surface of immune cells suggests that it can trigger cell reactions such as differentiation, activation, and proliferation [11]. It was shown that hLf accelerated differentiation of immature B and T cells and stimulated maturation of mouse splenic B cells [10,72]. Specific receptors nucleolin (expressed in B and T cells) and CD14 (expressed in monocytes) can be involved in triggering the cell response to stimulation by exogenous Lf. Since nucleolin is a multiligand protein acting as a shuttle between the cell surface and the nucleus [73,74], it can be expected that many biological functions of Lf are related to binding to this receptor.

## 5. Conclusions

The results of our study demonstrate positive pleiotropic effects of hLf on experimental animals subjected to sublethal irradiation that manifested in an increase in animal survival from 28% to 78%, a compensatory effect on radiation-induced body weight loss and the total number of leukocytes, changes in the differential leukocyte count, parameters of serum homeostasis, protective effect on the structural organization of the spleen, and normalization of the behavior of the irradiated mice. It should be noted that even a single injection of hLf immediately after irradiation was followed by the appearance of these positive effects. Our findings indicate the prospects for the development of radioprotective drugs based on this protein for the prevention and treatment of complications during occupational radiation exposure and for reducing side effects of radiation therapy.

## Figures and Tables

**Table 1:** Absolute number of leukocytes of different types in the blood of mice on day 3 after 7.5 Gy gamma irradiation (×10^6^/mL), Mean ± SEM.

Group	Day 3				
	Neutrophils	Lymphocytes	Monocytes	Eosinophils	Basophils
IR	0.24 ± 0.02 *	0.09 ± 0.02 *	0.06 ± 0.02 *	0.03 ± 0.01 *	0.07 ± 0.02 *
IR+Lf	0.41 ± 0.03 *	0.19 ± 0.03 *	0.07 ± 0.02 *	0.03 ± 0.01 *	0.07 ± 0.02 *
IR+Lf×2	0.39 ± 0.03 *	0.23 ± 0.01 *	0.06 ± 0.03 *	0.03 ± 0.01 *	0.05 ± 0.01 *
AC	1.85 ± 0.20	7.02 ± 0.39	0.80 ± 0.11	0.17 ± 0.03	0.97 ± 0.15
AC+Lf	2.38 ± 0.21	7.07 ± 0.41	1.33 ± 0.20	0.21 ± 0.05	1.21 ± 0.05
AC+Lf×2	2.01 ± 0.16	6.81 ± 0.11	1.00 ± 0.09	0.25 ± 0.04	1.12 ± 0.09

* *p* < 0.01 in comparison with the corresponding controls (Mann–Whitney U test).

**Table 2:** Absolute number of leukocytes of different types in the blood of mice on day 30 after 7.5 Gy gamma irradiation (×10^6^/mL), Mean ± SEM.

Group	Day 30				
	Neutrophils	Lymphocytes	Monocytes	Eosinophils	Basophils
IR	1.22 ± 0.12 *	2.54 ± 0.29 *	0.91 ± 0.32	0.13 ± 0.02	0.69 ± 0.12 *
IR+Lf	0.90 ± 0.10	4.05 ± 0.24 *	0.84 ± 0.16	0.18 ± 0.04	0.56 ± 0.05 *
IR+Lf×2	1.34 ± 0.11	5.79 ± 0.36	0.87 ± 0.16	0.14 ± 0.03	1.00 ± 0.16
AC	0.97 ± 0.08	6.90 ± 0.23	0.43 ± 0.09	0.16 ± 0.01	1.07 ± 0.13
AC+Lf	1.03 ± 0.14	5.91 ± 0.27	0.70 ± 0.13	0.14 ± 0.02	1.00 ± 0.08
AC+Lf×2	1.28 ± 0.17	6.06 ± 0.31	0.54 ± 0.16	0.22 ± 0.04	1.11 ± 0.05

* *p* < 0.01 in comparison with the corresponding controls (Mann–Whitney U test).

## Data Availability

Data are contained within the article.
